# Novel Loci for Non-Syndromic Coarctation of the Aorta in Sporadic and Familial Cases

**DOI:** 10.1371/journal.pone.0126873

**Published:** 2015-05-18

**Authors:** Julia Moosmann, Steffen Uebe, Sven Dittrich, André Rüffer, Arif B. Ekici, Okan Toka

**Affiliations:** 1 Friedrich-Alexander-Universität Erlangen-Nürnberg (FAU), Department of Pediatric Cardiology, Loschgestraße 15, 91054 Erlangen, Germany; 2 Friedrich-Alexander-Universität Erlangen-Nürnberg (FAU), Institute of Human Genetics, Schwabachanlage 10, 91054 Erlangen, Germany; 3 Friedrich-Alexander-Universität Erlangen-Nürnberg (FAU), Department of Pediatric Cardiac Surgery, Loschgestraße 15, 91054 Erlangen, Germany; Emory University School Of Medicine, UNITED STATES

## Abstract

**Backround:**

Coarctation of the aorta (CoA) accounts for 5-8% of all congenital heart defects. CoA can be detected in up to 20% of patients with Ullrich-Turner syndrome (UTS), in which a part or all of one of the X chromosomes is absent. The etiology of non-syndromic CoA is poorly understood. In the present work, we test the hypothesis that rare copy number variation (CNV) especially on the gonosomes, contribute to the etiology of non-syndromic CoA.

**Methods:**

We performed high-resolution genome-wide CNV analysis using the Affymetrix SNP 6.0 microarray platform for 70 individuals with sporadic CoA, 3 families with inherited CoA (n=13) and 605 controls. Our analysis comprised genome wide association, CNV burden and linkage. CNV was validated by multiplex ligation-dependent probe amplification.

**Results:**

We identified a significant abundance of large (>100 kb) CNVs on the X chromosome in males with CoA (p=0.005). 11 out of 51 (~ 22%) male cases had these large CNVs. Association analysis in the sporadic cohort revealed 14 novel loci for CoA. The locus on 21q22.3 in the sporadic CoA cohort overlapped with a gene locus identified in all familial cases of CoA (candidate gene *TRPM2*). We identified one CNV locus within a locus with high multipoint LOD score from a linkage analysis of the familial cases (*SEPT9*); another locus overlapped with a region implicated in Kabuki syndrome. In the familial cases, we identified a total of 7 CNV loci that were exclusively present in cases but not in unaffected family members.

**Conclusion:**

Of all candidate loci identified, the TRPM2 locus was the most frequently implicated autosomal locus in sporadic and familial cases. However, the abundance of large CNVs on the X chromosome of affected males suggests that gonosomal aberrations are not only responsible for syndromic CoA but also involved in the development of sporadic and non-syndromic CoA and their male dominance.

## Introduction

Recent studies of sporadic, non-syndromic congenital anomalies implicated rare *de novo* variants and copy number variation (CNV) as their etiology [1–3]. Several groups associated CNVs with the pathogenesis of congenital heart disease (CHD), including pulmonary atresia, tetralogy of Fallot, and left-sided outflow tract obstruction [4–8]. Left-sided outflow tract obstruction represents the most severe cardiac malformation syndrome, including bicuspid aortic valve (BAV), aortic valve stenosis (AS), coarctation of the aorta (CoA), and hypoplastic left heart syndrome (HLHS).

In this study we focus on CoA, which represents the third most frequent cardiac malformation with a prevalence of ~8% of all CHD. The Mendelian inheritance of CoA is very rare with only 1–2% of cases; in ~90% congenital CoA presents as a sporadic and non-syndromic congenital malformation [9, 10]. CoA can also be part of a syndrome, mainly the Ullrich-Turner syndrome (UTS) and the Kabuki syndrome (KS) [11, 12]. The prevalence of CoA in UTS is 7–18% and in KS 23% respectively [12–15]. The genetic basis of KS is poorly understood, except for cytogenetic abnormalities that have been associated with KS. In the majority of cases, changes on the gonosomes were identified, particularly on the X chromosome with ring X, monosomy X, and mosaic mutations; ring Y has also been associated with KS [16, 17]. Recently, a locus on 8p22-23.1 was implicated in to the pathogenesis of KS [18–22].

The most common chromosomal aberration in UTS is a complete monosomy X (50–75%) resulting from meiotic nondisjunction [23–25]. To a lesser degree, a partial or complete deletion of the short arm of the X chromosome has been implicated [23–27]. Strikingly, deletions of the short arm of the Y chromosome (Yp) can also lead to the complete phenotype of UTS including CoA [23, 24]. Such deletions usually involve the sex-determining region on Yp (SRY). Individuals with SRY deletions are clinically indistinguishable from female patients with UTS. This phenomenon has been attributed to haploinsufficiency of gonosomal homologue genes (GHG) that are non-recombinant gene pairs encoded on the X and Y chromosome analogous to autosomal gene pairs [23, 24, 28–30]. As GHG escape X-inactivation in females, two copies of each gene are expressed both in males and females [29]. Previously we hypothesized that loss-of-function mutations in selected GHG are involved in the development of non-syndromic CoA by a reduced gene dosage effect. We investigated a cohort of patients with sporadic CoA by a quite focused screening approach (gonosomal candidate gene approach) and identified transducin (beta)-like 1, Y-linked (*TBL1Y*), a gonosomal homologue gene associated with the Notch signaling pathway, to be involved in non-syndromic CoA by loss of function mutations[31]. In our current work we investigated a second and new cohort of 70 individuals with sporadic CoA and 3 families with inherited non-syndromic CoA by a wider screening concept (genome wide analyses of copy number variation). We tested the hypothesis that CNVs, particularly on the gonosomes, contribute not only to the development of syndromic CoA (like in UTS) but also to the development of non-syndromic sporadic CoA with male dominance.

## Material and Methods

### Ethics Statement

The study and particularly the collection and processing of the human tissue samples have been approved by the ethics committee of the University of Erlangen (Re.-No. 3818). Written informed consent was obtained from all participating individuals. All samples used have been de-identified for the study.

### Patient cohort

Our cohort included 70 individuals with isolated CoA (19 females; 51 males), 3 families (n = 13; 1 affected female, 5 affected males), and 605 controls; all individuals were of European genetic background. CoA was diagnosed by both echocardiography and aortic angiography. In some patients, an MR imaging study was also performed. Twenty-two individuals with CoA (31.4%) showed no other cardiac anomalies. Additional structural abnormalities in CoA including bicuspid aortic valve and septal defects are very common. Our cohort represents a typical distribution of concomitant structural abnormalities. Twenty-five (35.7%) of our patients had bicuspid aortic valve, 17 (24.2%) had a ventricular septal defect, 5 (7.1%) had persistent ductus arteriosus and 1 (1.4%) presented with Wolff Parkinson White syndrome, a common disorder of the conduction system. Individuals with genetically confirmed syndromic disorders, including UTS and KS, were excluded from the study. At the time of inclusion in our study, individuals were between 5 days and 48 years of age (average ~14.4 years) with a male-to-female ratio of 2.6 to 1. The control cohort included 605 individuals (382 males; 223 females) with no known heart disease. Genomic DNA was extracted from peripheral venous blood lymphocytes by QIAamp DNA Blood Kit (Qiagen). Quality assessments for DNA extraction and array preparation were performed before genotyping.

### DNA sequence variation and CNV genotyping

All sporadic cases and families with CoA (n = 83) were genotyped with the Affymetrix Genome-Wide Human SNPArray 6.0. single nucleotide polymorphisms (SNP) genotypes were called with the birdseed-v2 algorithm as implemented in the Affymetrix Power Tools. Segments with CNVs were called using birdseye, as implemented in the birdsuite software [32]. By implementation, this algorithm searches for novel, therefore rare CNV loci also outside known copy number polymorphism (CNP) sites. The same procedure was applied to all individuals from the 3 families with CoA. A group of 605 individuals with psoriatic arthritis, which had previously been genotyped using the procedure mentioned above, were used as controls [33]. A liftover to hg19 coordinates was carried out with the data from the 605 control individuals, as they had been genotyped using hg18 coordinates. At the beginning of birdsuite genotyping, individual arrays with poor quality were routinely removed by a standardized QC algorithm [32]. Segments from the sporadic cases and the 605 controls were then combined and translated into 379,666 pseudo-markers (start/endpoints of segments) to be used for association analysis using PLINK. For CNV burden analysis, the difference of the median and the minimum number of CNV segments within a cohort was added to the median, defining a cutoff value (For PSA, this was done after the liftover). All samples with more CNV segments than this cutoff value were excluded from the burden analysis. Both raw and processed copy number data haven been submitted to Gene Expression Omnibus (GEO) and are available through the accession number GSE67929 (sporadic cases) and GSE67930 (familial samples).

### Genome-wide SNP association

SNP genotypes, as determined by birdseed-v2 (part of the birdsuite algorithms), were filtered to exclude SNPs with more than 5% missing genotypes, a minor allele frequency of less than 1% and a marked deviation from Hardy-Weinberg equilibrium (HWE p-value < 0.001), reducing the original 909622 SNP markers to 722,556. A standard chi-square-based association analysis was then performed using PLINK.

### Linkage Analysis

We performed genome-wide linkage analysis in three non-related families. All DNA samples were genotyped with the Genome-Wide Human SNP Array 6.0 (Affymetrix, Santa Clara, Calif., USA). Genotypes were called by the Genotyping Console software v4.0 (Affymetrix). Simulation analysis for expected LOD scores (ELOD) for the given family pedigrees was performed with a parametric 2-point analysis with the software FastSLink [34]. Relationships of family members were verified by checking the genotype data with the software Graphical Representation of Relationships (GRR) [35]. Mendelian errors due to genotype errors were checked with the software Pedcheck and erroneous genotypes were removed from the data file [36]. Parametric multipoint linkage analysis and haplotype construction were done with the program Allegro [37–39]. Data handling was done with the software easyLinkage-Plus under the assumption of an autosomal-recessive and autosomal-dominant mode of inheritance with 100% penetrance, disease allele frequency of 0.01% and at least 0.01 cM intermarker distance [40]. Visualization of haplotype data was performed using the software haplopainter [41].

### MLPA validation of CNVs

All detected CNV regions were reviewed for gene content or gene proximity. From those we validated exemplarily eight region detected by SNP arrays by multiplex ligation dependent probe amplification (MLPA, MRC Holland) in up to 58 samples. We selected these regions according to technical MLPA assay design criteria. These eight regions contain the genes GSTM1, DEFB cluster on chromosome 8, NF1P2, ERVV1, FAM115C, SEPT9, TPTE and TRPM2, respectively. For all regions we could validate the copy number status in the analyzed samples except for the region containing NF1P2. In this region eight of 32 samples showed duplication instead of the deletion from the array data, possibly due to the highly variable pericentromeric structural variation region on chromosome 15q (S1 Table).

### Cardiac gene list

A cardiac gene list from CHDwiki was used to match with CNV loci from association analysis of sporadic cohort, overlapping regions from familial analysis and linkage regions. The cardiac gene list consists of 290 genes, which represents a set of most currently known gene-phenotypes linked with 139 cardiac defects. The use of this specialized ontology maximizes the relevance of the collected information to the CHD community and improves the consistency of this information. Each gene was reported to be involved in a cardiac phenotype in at least two independent publications [42].

## Results

### CNV burden analysis sporadic cohort

**Fig 1:**
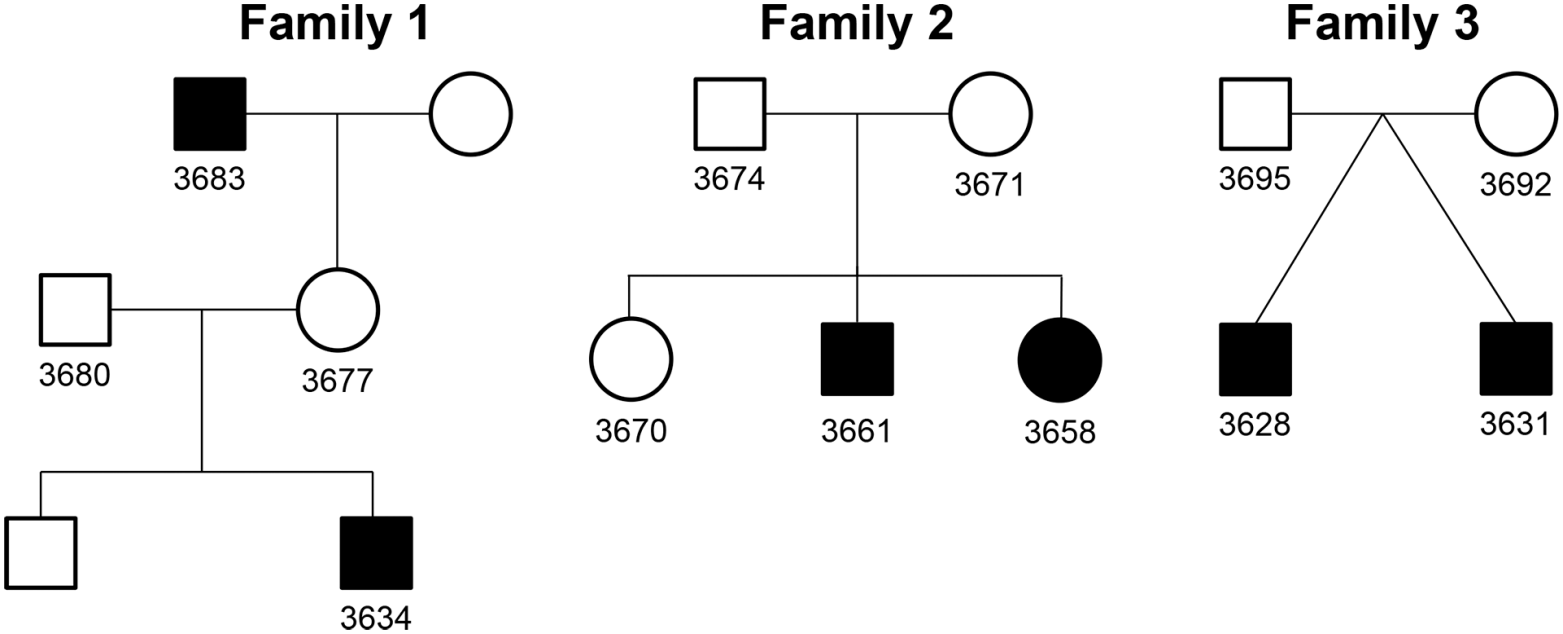
Family-tree of all families 1–3. Each family with 2 affected CoA patients. Family 1: grandfather 3683 and grandchild 3634; Family 2: patient 3661 and 3658. Sibling 3670 had a VSD; Family 3: dizygotic twins 3628 and 3631.

We included 70 individuals with sporadic CoA and 490 of the 605 PSA controls, as well as 3 families with 2 affected individuals per family with CoA (Fig 1) for a separate burden analysis in familial cases. All samples met in-house quality criteria. Overall, we detected 7,160 CNVs with >10kb (3,127 deletions and 4,033 duplications) in CoA individuals and a total of 17,990 CNVs larger than 10kb (8,600 deletions and 9,390 duplications) in the control cohort. We determined a size threshold at 100kb and distinguished between autosomes and gonosomes. The optimal size threshold for this type of analysis has been demonstrated previously to be around 100kb [43]. Ninety-three CNVs > 100kb were identified in CoA (~1.33/individual) and 2,151 in the control cohort (~4.39/individual). We then tested for a possible abundance of large CNVs in cases versus controls, both in autosomes and on the X chromosome, the latter being tested separately for males (definition of a CNV to have a copy number count other than one and be outside of the pseudo-autosomal regions) and females (definition of a CNV as in autosomes). Hereby we tested for a higher proportion of large CNV segments which might be present in cases compared to controls, as shown previously in a study concerning the genetics of short stature [43]. Addressing our hypothesis that CNVs particularly on the gonosomes contribute to sporadic and non-syndromic CoA we were able to identify a significant abundance (p = 0.005, with a Bonferroni corrected significance threshold of 0.0167) of large (>100 kb) X-chromosomal CNVs in males by applying a one-sided Fisher’s exact test with the alternative hypothesis being an odds ratio greater than 1 (Table 1). Of the 51 male cases, 11 had CNV segments >100kb on the X chromosome. Just a subset of this CNV segments are localized in coding regions. Genes contained within these CNV segments are *SPACA5*, *ZNF630*, *SSX6* (all Xp11.23) and *PCDH11X* (Xq21.3). Comparison of autosomal CNV burden in cases versus controls showed no significant enrichment of large CNVs in cases.

**Table 1:** CNV burden analysis of large (>100 kb) CNVs on gonosomes and autosomes in the sporadic CoA cohort.

	CoA			Controls			OR	p-value for CNVs >100kb
	> 10kb	< 100kb	> 100kb	> 10kb	<100kb	> 100kb		
**CNV X-Male**	677	663	14	4,187	4,152	35	2.5	**0.005**
**CNV X-Female**	41	39	2	284	253	31	0.40	0.94
**CNV Autosomes**	6,442	5,671	77	13,519	11,434	2,085	0.07	≈1

Total number of CNVs on gonosomes and autosomes. CNV burden was significantly higher for large (>100kb) CNVs in male patients (p<0.01) on the X-chromosome. No changes were seen for the X-chromosome in females and on the autosomes. A one-sided Fisher‘s exact test was calculated from a contingency table with the total number of CNV segments, with a size between 10 and 100 kb and those larger than 100 kb in both cases and controls.

### CNV association analysis in sporadic samples

The pseudo-markers from the 70 sporadic cases and 605 control individuals were tested for association using a one-sided permutation-based approach as implemented in PLINK, with 100,000 permutations. Only segments with at least 5 markers and spanning 10 kb or more were evaluated. Of the associated loci, 5 exceeded the significance threshold of 5E-05 as deletions and 9 as duplications (Table 2). The duplication locus on chromosome 17 did not quite pass the significance threshold, but was retained due to overlap with a linkage region (Table 3). The DEFB4 cluster on chromosome 8 is a known CNP locus also present in PSA cases. Since one-sided tests were used, and due to the previous literature (see discussion), this locus was also retained. X-chromosomal association analysis was carried out for males and females separately, but showed no significantly associated loci.

**Table 2:** CNV Loci in the sporadic CoA cohort.

Location	Start	Length (bp)	CN	Probands (n = 70)	Controls (n = 605)	p-value	OR	Genes
1p13.3	110,224,384	18,569	Loss	43	103	1.0E-5	7.76	GSTM2, GSTM1
6p22.1	29,837,314	211	Loss	37	119	5.0E-5	4.58	None
6p25.3	833,041	9,659	Gain	5	0	2.0E-05	89.11	None
7q35	143,246,369	286,629	Gain	9	0	1.0E-5	12.35	CTAGE15P,FAM115C,CTAGE6P
8p23.3	2,130,233	12,694	Gain	6	0	1.0E-5	12.68	None
8p23.1	7,238,020	549,942	Loss	9	1	1.0E-5	N/A	DEFB4B, DEFB103A, SPAG11B, DEFB104A, DEFB106A, DEFB105A, DEFB107A, SPAG11A, DEFB4A
8p23.1	7,250,380	549,010	Gain	11	0	1.0E-5	N/A	DEFB4B, DEFB103A, SPAG11B, DEFB104A, DEFB106A, DEFB105A, DEFB107A, SPAG11A, DEFB4A
15q11.2	20,830,374	1,109,449	Loss	11	9	1.0E-5	N/A	POTEB
15q11.1	20,627,493	1,415,261	Gain	14	23	1.0E-5	N/A	GOLGA6L6, POTEB, GOLGA6L6
16p11.2	32,564,512	44,762	Gain	8	3	1.0E-5	6.33	None
17q25.3	75,343,091	58,545	Gain	4	1	6.7E-4	25.89	SEPT9
17p11.2	18,412,559	52,662	Gain	7	1	1.0E-5	36.61	None
19q13.41	53,524,199	28,030	Loss	58	167	1.0E-5	67.11	None
21p11.2	10,898,194	16,632	Gain	5	0	2.0E-5	N/A	TPTE
21q22.3	45,827,393	13,027	Gain	6	1	3.0E-5	56.63	TRPM2

14 loci exceeded the significance threshold of 5 E-05, including 5 deletions and 9 duplications. The first locus on chromosome 17 did not quite pass the significance threshold, but was retained due to overlap with a linkage region.

**Table 3:** Match between linkage loci and genes of cardiac gene list.

		Parametric multipoint HLOD scores	SNP marker		genetic map coordinates (cM), deCode-2007		physical map coordinates (bp), hg19			
Locus	Chr		start	stop	Start	stop	start	stop	Locus size (bp)	Cardiac Genes in Locus
1	1	1.62	rs12057617	rs10911529	151.71	185.58	157,948,380	184,187,068	26,238,689	PRRX1, TNR, LAMC1
2	2	1.62	rs441469	rs3856454	1.13	22.43	129,027	8,791,510	8,662,484	
3	2	1.62	rs853778	rs3754750	174.23	179.83	169,811,223	173,887,052	4,075,830	
4	3	1.62	rs9881298	rs6796856	216.08	221.16	194,573,238	197,447,653	2,874,416	
5	5	1.62	rs160725	rs200841	5.58	18.17	2,309,192	6,737,133	4,427,942	
6	5	1.62	rs13177282	rs299915	48.98	74.72	30,207,797	58,123,361	27,915,565	GDNF, FGF10, ISL1
7	5	1.62	rs306577	rs4703270	107.17	112.18	94,052,732	103,263,133	9,210,402	
8	5	1.62	rs17075700	rs6874441	188.22	188.63	172,635,139	172,769,948	134,810	NKX2-5
9	6	1.62	rs9384603	rs9496709	109.76	146.48	106,788,485	143,939,012	37,150,528	SESN1, GJA1, HEY2, LAMA2, TCF21, CITED2
10	6	1.62	rs4708721	rs12189695	186.18	189.68	168,729,951	170,891,042	2,161,092	DLL1
11	7	1.62	rs10254065	rs1006622	42.59	50.58	26,388,551	31,108,895	4,720,345	HOXA1, JAZF1
12	8	1.62	rs12681862	rs1434757	77.48	78.51	68,041,789	69,042,584	1,000,796	
13	9	1.62	rs2000289	rs17352208	73.92	78.25	79,450,929	83,633,017	4,182,089	
14	12	1.62	rs839738	rs11060968	152.88	166.83	128,021,206	131,006,546	2,985,341	
15	13	1.59	rs7995874	rs9512560	16.99	18.14	27,172,745	27,731,793	559,049	
16	17	1.62	rs8071099	rs920130	80.11	118.97	52,137,104	75,527,911	23,390,808	TBX2, ITGB4
17	21	1.62	rs197576	rs7283702	13.00	16.25	19,348,804	20,357,111	1,008,308	
18	X	1.20	rs7050735	rs3788760	17.92	35.00	9,602,273	21,740,301	12,138,029	MID1, RBBP7, RAI2
19	X	1.20	rs768272	rs5918011	48.87	62.78	32,254,551	40,473,965	8,219,415	BCOR

Of the significant 19 linkage loci of familial cases, 9 loci contained genes (n = 22) of the cardiac gene list.

### SNP analysis in sporadic CoA cohort

A SNP association using 722,556 SNPs in the unrelated cases and controls was performed, and showed no evidence of association past the genome-wide significance threshold (data not shown).

### CNV burden in familial cases

CNV burden analysis for large CNVs >100 kb was performed analogous to the analysis in sporadic cases. It revealed no difference in gonosomal CNV burden. However, a difference was seen for the autosomes (Table 4). Familial CoA cases showed significantly more large deletions >100 kb (p<0.01) and less large duplications >100 kb (p<0.01) compared to controls.

**Table 4:** CNV burden analysis of large (>100kb) CNVs on gonosomes and autosomes in familial cases versus healthy individuals.

	CoA			Controls			OR	p-value for CNVs >100kb
	> 10kb	< 100kb	> 100kb	> 10kb	<100kb	> 100kb		
**Deletion Autosomes**	217	172	45	221	196	25	2.05	**<0.01**
**Duplication Autosomes**	1,003	949	54	191	151	40	0.21	**<0.01**
**CNV-X-Male**	86	85	1	54	54	0		1
**CNV-X-Female**	8	8	0	3	3	0		1

CNV burden in familial CoA was significantly higher for deletions >100kb (p<0.01) and significantly lower for duplications > 100 kb (p<0.01). The total number of CNVs were higher in controls. No significant difference was observed between males and females. Fisher ‘s exact test was calculated from a contingency table with the total number of CNV segments, with a size between 10 and 100 kb and those larger than 100 kb in both cases and controls.

### Linkage analysis in families with CoA

**Fig 2:**
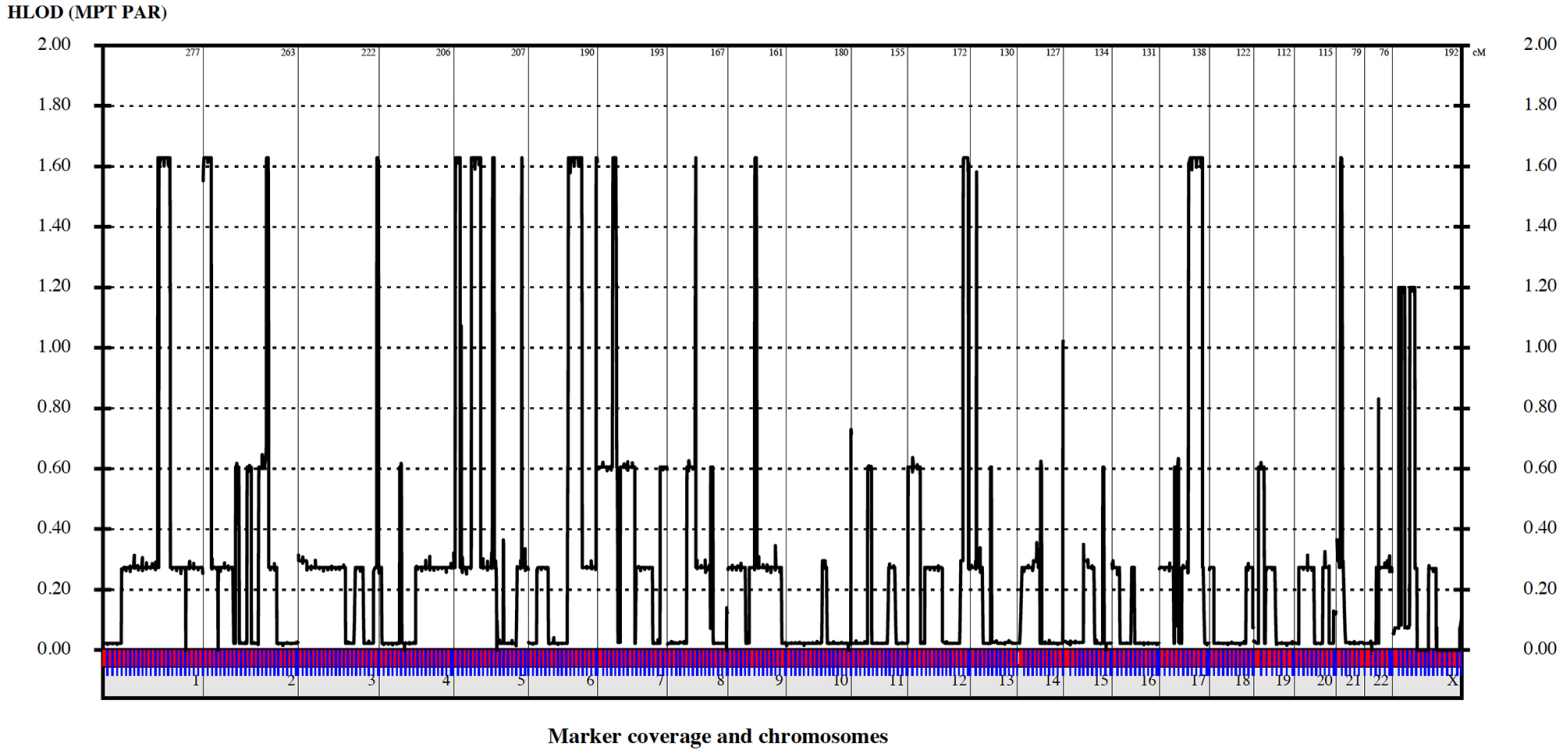
LOD-Plot showing genome wide linkage analysis in all three families. 19 linkage regions with a maximum LOD-score of 1.62 were identified, which are illustrated by multipoint LOD scores above background.

Even though we did not expect to achieve significant LOD scores due to small pedigrees, we performed a linkage analysis. 521,435 SNPs with perfect call rate were used in a multipoint linkage analysis assuming dominant or recessive inheritance for all pedigrees, producing a union of all regions with a LOD score raising above the background. This approach yielded a total of 19 linkage regions with LOD scores of up to 1.62 (Fig 2).

### CNV analysis in families with CoA

CNV calling with the birdseye software produced 1783 CNV segments larger than 10 kb (444 deletions, 1339 duplications), which were checked for overlap in the affected 6 individuals of the 3 families. One duplication locus was present in all 6 affected individuals but not in the unaffected controls, one locus in 5 cases / 0 controls and 5 loci with 4 cases / 0 controls (Table 5). The duplication locus on chromosome 6 shared by 4 cases and 0 controls partially overlaps with a linkage region.

**Table 5:** Overlapping CNV segments in familial CoA with candidate gene.

Location	Start	Length (bp)	CN	Affected/Unaffected	Genes
2p16.1	55,167,820	8,292	Gain	4/0	EML6
6p21.1	41,904,483	777	Gain	4/0	CCND3
11q13.1	65,278,385	24,056	Gain	4/0	SCYL1
19q13.2	42,385,629	20,274	Gain	4/0	ARHGEF1
21q22.3	45,812,755	14,638	Gain	6/0	TRPM2
22q13.1	38,510,598	14,141	Gain	5/0	PLA2G6
22q12.2	31,474,554	10,792	Gain	4/0	SMTN

Seven overlapping CNVs shared in >4 individuals with CoA were identified. The chromosome 21 CNV locus was present in all familial CoA cases. It contains *TRPM2*, which was also identified in the sporadic CoA cohort.

### Cardiac gene list

A list with 290 cardiac candidate genes was compared with all CNV segments of the sporadic and familial CoA cohort as well as the linkage regions of the familial cases [42].

An overlap between calculated linkage regions with this cardiac gene list identified 22 cardiac genes located within the linkage regions as novel candidate genes for CoA (Table 3).

## Discussion

Due to the high incidence of CoA in UTS an KS patients and the male dominance of CoA in non-syndromic sporadic cases we previously screened for mutation in GHG by a gonosomal candidate gene approach[31]. To follow up on our idea of gonosomal abnormalities in sporadic non-syndromic CoA we now extended our mutation detection from single gene analyses (candidate gene approach by Sanger sequencing) to a genome wide CNV analysis. Our hypothesis was to evaluate whether CNV, particularly on the gonosomes, contribute to the development of sporadic and non-syndromic CoA.

**Fig 3:**
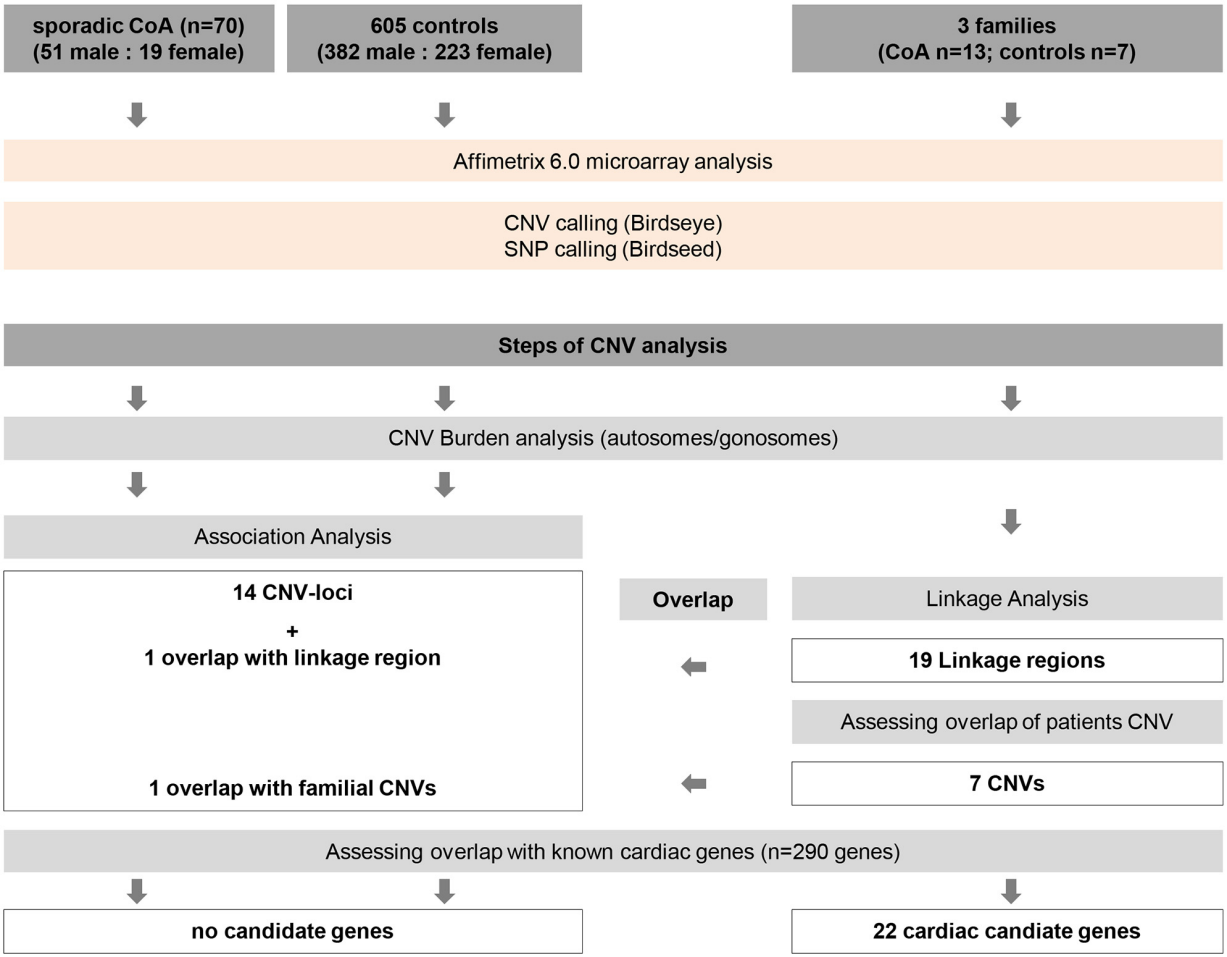
Overview of workflow of CNV analysis in sporadic CoA patients and families with CoA. Genotyping was performed in 70 individuals with sporadic CoA, 605 healthy controls, and 3 families with 6 CoA cases and 7 healthy individuals. CNV calling was performed using Birdseye and Birdseed software. CNV burden analysis in sporadic cohort and familial CoA samples was performed for autosomes and gonosomes separately. Association analysis was performed in the sporadic CoA cohort and revealed 14 CNV loci. Overlap of CNVs in familial patients was assessed and revealed 7 CNVs shared in >4 patients, overlapping with one CNV locus in the sporadic CoA cohort. Linkage analysis in familial samples revealed 19 linked regions, one overlapping with the CNV locus on chromosome 17, which did not pass the threshold of significance in the association analysis.

CNV have been associated with the pathogenesis of complex congenital heart disease and left sided outflow tract obstruction [5–8]. To our knowledge our study is the first genome wide CNV analysis in CoA cohorts. Our comprehensive genotype analyses contained sporadic and familial cases with CoA and addressed gender- specific genetic modifier. We performed CNV association and burden analysis. CNV burden was evaluated after assigning a size threshold of >100kb and distinguishing between autosomes and gonosomes. Analysis was performed separately for sporadic CoA cases versus controls and familial CoA cases vs. healthy family members. Gonosomes were tested separately for males and females. Additionally, a list with 290 cardiac candidate genes was compared with all CNV segments of the sporadic and familial CoA cohorts as well as the linkage regions of the familial cases to assess the overlap of gene content (Fig 3).

Regarding the CNV burden in the group of sporadic CoA cases, a significant gonosomal abundance (p = 0.005) for large X-chromosomal CNVs (>100 kb) in males was observed. This abundance of large X-chromosomal CNVs in sporadic male CoA cases can be correlated with the clinical finding of a 2:1 male-to-female incidence in CoA. Males cannot compensate for alterations of X-chromosomal gene dosage. Only a subset of the X-chromosomal CNV segments were within coding regions of chromosome X. However, we still have limited knowledge on intronic regulators of gene activity, particularly on the X chromosome. We could identify 4 genes within these chromosomal segments; *SPACA5* (Sperm Acrosome-Associated Protein 5), *ZNF630* (zinc finger protein 630), *SSX6* (synovial sarcoma, X breakpoint 6), and *PCDH11X* (protocadherin 11 X-linked). In a comparative genomic hybridization (CGH) array analysis a duplication of *SPACA5* (and *ZNF81/ZNF182*) has been described in a boy with developmental delay, autistic features, and growth and speech delay [44]. *SSX6* belongs to the *SSX* gene family, whereas *SSX1*, *2* and *4* have found to be involved in synovial sarcomas, *SSX6* is expressed in melanoma cell lines [45]. *ZNF630* resides on an area of chromosome X that hast been implicated in non-syndromic X-linked mental retardation [46]. *PCDH11X* belongs to the protocadherin gene family of calcium-dependent cell adhesion and recognition proteins and is located in a major X/Y block of homology [47]. The PCDH11X protein is thought to play a fundamental role in cell-cell recognition essential for the segmental development of the central nervous system. Alternative splicing in *PCDH11X* results in multiple transcript variants. It has been speculated as a potential candidate gene for late-onset Alzheimer disease [48, 49]. However, the main interacting partners and pathways of PCDH11X as well as its distinct genotype-phenotype correlations are unknown.

Contrary to the sporadic cases of CoA, the CNV burden for gonosomes was not different in familial cases. Here we could identify a significantly different CNV burden for the autosomes, with more deletions and less duplications on autosomes in the familial cases of CoA. This different pattern of CNV burden may indicate that the pathogenesis of sporadic and familial cases could be different. However, the number of families we investigated in this study was small compared to the number of patients in the sporadic cohort.

In the association analyses of the sporadic cohort we identified 14 CNV loci (threshold of 5E-05) derived from segments in 5–58 of 70 patients, which are mostly novel and therefore not common copy number variations (CNPs). One region on chromosome 8p23.1 was deleted in 9 patients and strikingly the same region was duplicated in 11 patients, only one control showed a deletion in this area. The ratio of the CoA subgroups (CoA only, CoA with BAV or VSD) within those 20 individuals did not significantly differ from the primary cohort of 70 individuals although the majority of patients had isolated CoA (10 patients = 50% with CoA only, 7 patients = 35% with additional BAV, 3 = 15% with additional VSD, see Table 6). This locus contains the genes *DEFB4B*, *DEFB103A*, *SPAG11B*, *DEFB104A*, *DEFB106A*, *DEFB105A*, *DEFB107A*, *SPAG11A*, *DEFB4A*. Various studies described copy number changes at this locus in patients with KS or Kabuki-like syndromes’ some of these patients have been reported to have hypoplastic left ventricle and aorta. However, whether copy number changes on 8p22–23.1 are associated with KS or not is debated in the literature [18–22, 50]. Hypoplastic aortic arch or CoA is only an intermediate phenotype of KS. In our analyses 20 individuals of the 70 sporadic cases with CoA had large CNVs on 8p22–23.1. Thus, we suggest that the region 8p22–23.1 is a candidate region for CoA regardless of additional phenotypic features of KS. While we are aware of the significance of this locus for psoriasis and psoriatic arthritis[51], we have used one-sided tests throughout, therefore our findings do not reflect any CNVs possibly present in the PSA controls, but instead have a higher relative abundance in our patients.

**Table 6:** Distribution of CNV on 8p23.1 in all affected patients according to their phenotype.

Locus 8p23.1	Isolated CoA	CoA+BAV	CoA+VSD
Deletion (n = 9)	3	4	2
Duplication (n = 11)	7	3	1

The 20 patients with CNV on 8p23.1 represent a typical distribution of concomitant malformation in CoA. 10 patients with isolated CoA, 7 with additional BAV and 3 with additional VSD.

The CNV analyses in the familial cases revealed 7 rare (non-CNP) CNVs, which overlapped within affected individuals from all 3 families. One CNV locus (*TRPM2*; transient receptor potential cation channel, subfamily M, member 2) was present in all 6 affected individuals of the 3 CoA families and absent in all unaffected individuals. Another CNV locus (*PLA2G6*, phospholipase A2, group VI) was present in 5 of 6 familial CoA cases and none of the unaffected individuals. Five CNV loci (*SMTN*, smoothelin; *ARHFEF1*, Rho guanine nucleotide exchange factor (GEF) 1; *SCYL1*, S. cerevisiae1-like1; *CCND3*, cyclin d3; *EML6*, echinoderm microtubule associated protein like 6) were present in 4 of 6 familial CoA cases and none of the unaffected individuals (Table 5). Our association analyses considered sporadic cases only due to the limited number of familial cases. However, we tested for overlap between the groups, comparing association loci of sporadic CoA cases with overlapping loci of familial CoA cases,—and with the linkage regions of the familial CoA cases.

The CNV locus of *TRPM2* was not only detected in all 6 affected individuals of the familial CoA cases but also in 6 individuals of the sporadic CoA cohort, providing us a potentially important new candidate or modifier genes for the CoA phenotype. TRPM2 is a well-known oxidant-sensitive Ca2+ permeable channel implicated in mediating endothelial apoptosis and in promoting vascular injury and inflammation [52]. TRPM2 also contributes to production of vasoactive nitric oxide via the p38/JNK pathway [53]. In an established mouse model, cardiac TRPM2 channel activity protected the heart from ischemia/reperfusion injury by ameliorating mitochondrial dysfunction and reducing reactive oxygen species levels [54]. So far there are no data on alteration of TRPM2 activity in a model of endovascular stenosis or even coarctation of the aortic isthmus. Endovascular stenosis can augment sheer stress-induced endothelial cell apoptosis and inflammation [55]. The cause of CoA is certainly heterogeneous, polygenic and pleiotropic. Altered oxidant-sensitive Ca2+ permeable channel activity involved in endovascular apoptosis and inflammation and regulation of vascular tone via nitric oxide may represent a component of the primary structural defect in CoA. The TRMP2 pathway could also be only an associated component for vascular stiffness and progressive arterial hypertension in CoA patients without involvement in this etiology. Further studies are needed.

Due to the limited number of familial cases, we decided to decrease the threshold for relevant linkage loci and highlighted in a first step all loci above background. The analyses revealed 19 linkage regions with LOD scores of up to 1.62 (Fig 2). We filtered the number of candidate genes by matching them with a list of known cardiac genes involving 139 cardiac defects. Of these 19 loci, 9 contained 22 overlapping cardiac genes listed in Table 3.

## Conclusion

Our study provides new insight into the genetic basis of non-syndromic CoA by identifying new candidate genes through CNV association and burden analysis. Of all candidate loci identified, the TRPM2 gene locus was the most frequently implicated autosomal locus in both, sporadic and familial cases. The abundance of large CNVs on the X-Chromosome of affected males, suggests that gonosomal aberrations are not only involved in the development of syndromic CoA, like in UTS or KS, but also likely responsible for sporadic and non-syndromic CoA and the male dominance observed in this malformation. Further studies are needed and subject to our current efforts to better understand the genetic but also the epigenetic factors leading to CoA.

## Supporting Information

S1 Table MLPA validation From all detected CNVs eight with gene content were chosen for validation with MLPA. Only those DNA samples with an aberrant CNV in the particular region were analyzed with a MLPA assay. All CNVs from the microarray analysis could be validated (+), except for the CNV status of the NF1P2 region in eight of 32 samples (duplications instead of deletions); n/a = not applicable because of absent CNV in microarray data.
(DOCX)

## References

[1] Younkin SG, Scharpf RB, Schwender H, Parker MM, Scott AF, Marazita ML, et al A genome-wide study of de novo deletions identifies a candidate locus for non-syndromic isolated cleft lip/palate risk. BMC genetics. 2014;15:24 10.1186/1471-2156-15-24 24528994 PMC3929298

[2] Lindstrand A, Davis EE, Carvalho CM, Pehlivan D, Willer JR, Tsai IC, et al Recurrent CNVs and SNVs at the NPHP1 locus contribute pathogenic alleles to Bardet-Biedl syndrome. American journal of human genetics. 2014;94(5):745–54. 10.1016/j.ajhg.2014.03.017 24746959 PMC4067552

[3] Du X, An Y, Yu L, Liu R, Qin Y, Guo X, et al A genomic copy number variant analysis implicates the MBD5 and HNRNPU genes in Chinese children with infantile spasms and expands the clinical spectrum of 2q23.1 deletion. BMC medical genetics. 2014;15:62 10.1186/1471-2350-15-62 24885232 PMC4061518

[4] Soemedi R, Wilson IJ, Bentham J, Darlay R, Topf A, Zelenika D, et al Contribution of global rare copy-number variants to the risk of sporadic congenital heart disease. American journal of human genetics. 2012;91(3):489–501. 10.1016/j.ajhg.2012.08.003 22939634 PMC3511986

[5] Greenway SC, Pereira AC, Lin JC, DePalma SR, Israel SJ, Mesquita SM, et al De novo copy number variants identify new genes and loci in isolated sporadic tetralogy of Fallot. Nature genetics. 2009;41(8):931–5. 10.1038/ng.415 19597493 PMC2747103

[6] Xie L, Chen JL, Zhang WZ, Wang SZ, Zhao TL, Huang C, et al Rare de novo copy number variants in patients with congenital pulmonary atresia. PloS one. 2014;9(5):e96471 10.1371/journal.pone.0096471 24826987 PMC4020819

[7] Silversides CK, Lionel AC, Costain G, Merico D, Migita O, Liu B, et al Rare copy number variations in adults with tetralogy of Fallot implicate novel risk gene pathways. PLoS genetics. 2012;8(8):e1002843 10.1371/journal.pgen.1002843 22912587 PMC3415418

[8] Hitz MP, Lemieux-Perreault LP, Marshall C, Feroz-Zada Y, Davies R, Yang SW, et al Rare copy number variants contribute to congenital left-sided heart disease. PLoS genetics. 2012;8(9):e1002903 10.1371/journal.pgen.1002903 22969434 PMC3435243

[9] Stoll C, Alembik Y, Dott B. Familial coarctation of the aorta in three generations. Annales de genetique. 1999;42(3):174–6. 10526662

[10] Gerboni S, Sabatino G, Mingarelli R, Dallapiccola B. Coarctation of the aorta, interrupted aortic arch, and hypoplastic left heart syndrome in three generations. Journal of medical genetics. 1993;30(4):328–9. 8487284 10.1136/jmg.30.4.328 PMC1016347

[11] Wellesley DG, Slaney S. Kabuki make-up and Turner syndromes in the same patient. Clinical dysmorphology. 1994;3(4):297–300. 7894734

[12] Digilio MC, Marino B, Toscano A, Giannotti A, Dallapiccola B. Congenital heart defects in Kabuki syndrome. American journal of medical genetics. 2001;100(4):269–74. 11343317 10.1002/ajmg.1265

[13] Sybert VP. Cardiovascular malformations and complications in Turner syndrome. Pediatrics. 1998;101(1):E11 9417175 10.1542/peds.101.1.e11

[14] Volkl TM, Degenhardt K, Koch A, Simm D, Dorr HG, Singer H. Cardiovascular anomalies in children and young adults with Ullrich-Turner syndrome the Erlangen experience. Clinical cardiology. 2005;28(2):88–92. 15757080 10.1002/clc.4960280209 PMC6654047

[15] Gotzsche CO, Krag-Olsen B, Nielsen J, Sorensen KE, Kristensen BO. Prevalence of cardiovascular malformations and association with karyotypes in Turner's syndrome. Archives of disease in childhood. 1994;71(5):433–6. 7826114 10.1136/adc.71.5.433 PMC1030059

[16] McGinniss MJ, Brown DH, Burke LW, Mascarello JT, Jones MC. Ring chromosome X in a child with manifestations of Kabuki syndrome. American journal of medical genetics. 1997;70(1):37–42. 9129739

[17] Stankiewicz P, Thiele H, Giannakudis I, Schlicker M, Baldermann C, Kruger A, et al Kabuki syndrome-like features associated with a small ring chromosome X and XIST gene expression. American journal of medical genetics. 2001;102(3):286–92. 11484209 10.1002/ajmg.1462

[18] Milunsky JM, Huang XL. Unmasking Kabuki syndrome: chromosome 8p22–8p23.1 duplication revealed by comparative genomic hybridization and BAC-FISH. Clinical genetics. 2003;64(6):509–16. 14986831 10.1046/j.1399-0004.2003.00189.x

[19] Shieh JT, Hudgins L, Cherry AM, Shen Z, Hoyme HE. Triplication of 8p22–8p23 in a patient with features similar to Kabuki syndrome. American journal of medical genetics Part A. 2006;140(2):170–3. 10.1002/ajmg.a.31036 16353235

[20] Hoffman JD, Zhang Y, Greshock J, Ciprero KL, Emanuel BS, Zackai EH, et al Array based CGH and FISH fail to confirm duplication of 8p22-p23.1 in association with Kabuki syndrome. Journal of medical genetics. 2005;42(1):49–53. 10.1136/jmg.2004.024372 15635075 PMC1735911

[21] Turner C, Lachlan K, Amerasinghe N, Hodgkins P, Maloney V, Barber J, et al Kabuki syndrome: new ocular findings but no evidence of 8p22-p23.1 duplications in a clinically defined cohort. European journal of human genetics: EJHG. 2005;13(6):716–20. 10.1038/sj.ejhg.5201377 15785777

[22] Sanlaville D, Genevieve D, Bernardin C, Amiel J, Baumann C, de Blois MC, et al Failure to detect an 8p22–8p23.1 duplication in patients with Kabuki (Niikawa-Kuroki) syndrome. European journal of human genetics: EJHG. 2005;13(5):690–3. 10.1038/sj.ejhg.5201383 15770228

[23] Marino B, Digilio MC. Congenital heart disease and genetic syndromes: specific correlation between cardiac phenotype and genotype. Cardiovascular pathology: the official journal of the Society for Cardiovascular Pathology. 2000;9(6):303–15. 11146300 10.1016/s1054-8807(00)00050-8

[24] Ranke MB, Saenger P. Turner's syndrome. Lancet. 2001;358(9278):309–14. 10.1016/S0140-6736(01)05487-3 11498234

[25] Lacka K. [Turner's syndrome—correlation between karyotype and phenotype]. Endokrynologia Polska. 2005;56(6):986–93. 16821224

[26] Boucher CA, Sargent CA, Ogata T, Affara NA. Breakpoint analysis of Turner patients with partial Xp deletions: implications for the lymphoedema gene location. Journal of medical genetics. 2001;38(9):591–8. 11546827 10.1136/jmg.38.9.591 PMC1734929

[27] Ogata T, Muroya K, Matsuo N, Shinohara O, Yorifuji T, Nishi Y, et al Turner syndrome and Xp deletions: clinical and molecular studies in 47 patients. The Journal of clinical endocrinology and metabolism. 2001;86(11):5498–508. 10.1210/jcem.86.11.8058 11701728

[28] Tzancheva M, Kaneva R, Kumanov P, Williams G, Tyler-Smith C. Two male patients with ring Y: definition of an interval in Yq contributing to Turner syndrome. Journal of medical genetics. 1999;36(7):549–53. 10424817 PMC1734411

[29] Lahn BT, Page DC. Functional coherence of the human Y chromosome. Science. 1997;278(5338):675–80. 9381176 10.1126/science.278.5338.675

[30] Ogata T, Matsuo N. Sex chromosome aberrations and stature: deduction of the principal factors involved in the determination of adult height. Human genetics. 1993;91(6):551–62. 8340109 10.1007/BF00205079

[31] Tagariello A, Breuer C, Birkner Y, Schmidt S, Koch AM, Cesnjevar R, et al Functional null mutations in the gonosomal homologue gene TBL1Y are associated with non-syndromic coarctation of the aorta. Current molecular medicine. 2012;12(2):199–205. 22280357 10.2174/156652412798889027

[32] Korn JM, Kuruvilla FG, McCarroll SA, Wysoker A, Nemesh J, Cawley S, et al Integrated genotype calling and association analysis of SNPs, common copy number polymorphisms and rare CNVs. Nature genetics. 2008;40(10):1253–60. 10.1038/ng.237 18776909 PMC2756534

[33] Huffmeier U, Uebe S, Ekici AB, Bowes J, Giardina E, Korendowych E, et al Common variants at TRAF3IP2 are associated with susceptibility to psoriatic arthritis and psoriasis. Nature genetics. 2010;42(11):996–9. 10.1038/ng.688 20953186 PMC2981079

[34] Weeks DE, Lehner T, Squires-Wheeler E, Kaufmann C, Ott J. Measuring the inflation of the lod score due to its maximization over model parameter values in human linkage analysis. Genetic epidemiology. 1990;7(4):237–43. 10.1002/gepi.1370070402 2227370

[35] Abecasis GR, Cherny SS, Cookson WO, Cardon LR. GRR: graphical representation of relationship errors. Bioinformatics. 2001;17(8):742–3. 11524377 10.1093/bioinformatics/17.8.742

[36] O'Connell JR, Weeks DE. PedCheck: a program for identification of genotype incompatibilities in linkage analysis. American journal of human genetics. 1998;63(1):259–66. 10.1086/301904 9634505 PMC1377228

[37] Gudbjartsson DF, Jonasson K, Frigge ML, Kong A. Allegro, a new computer program for multipoint linkage analysis. Nature genetics. 2000;25(1):12–3. 10.1038/75514 10802644

[38] Gudbjartsson DF, Thorvaldsson T, Kong A, Gunnarsson G, Ingolfsdottir A. Allegro version 2. Nat Genet. 2005;37(10):1015–6. 10.1038/ng1005-1015 16195711

[39] Greenberg DA, Abreu PC. Determining trait locus position from multipoint analysis: accuracy and power of three different statistics. Genetic epidemiology. 2001;21(4):299–314. 10.1002/gepi.1036 11754466

[40] Hoffmann K, Lindner TH. easyLINKAGE-Plus—automated linkage analyses using large-scale SNP data. Bioinformatics. 2005;21(17):3565–7. 10.1093/bioinformatics/bti571 16014370

[41] Thiele H, Nurnberg P. HaploPainter: a tool for drawing pedigrees with complex haplotypes. Bioinformatics. 2005;21(8):1730–2. 10.1093/bioinformatics/bth488 15377505

[42] Barriot R, Breckpot J, Thienpont B, Brohee S, Van Vooren S, Coessens B, et al Collaboratively charting the gene-to-phenotype network of human congenital heart defects. Genome medicine. 2010;2(3):16 10.1186/gm137 20193066 PMC2873794

[43] Zahnleiter D, Uebe S, Ekici AB, Hoyer J, Wiesener A, Wieczorek D, et al Rare copy number variants are a common cause of short stature. PLoS genetics. 2013;9(3):e1003365 10.1371/journal.pgen.1003365 23516380 PMC3597495

[44] Alesi V, Bertoli M, Barrano G, Torres B, Pusceddu S, Pastorino M, et al 335.4 kb microduplication in chromosome band Xp11.2p11.3 associated with developmental delay, growth retardation, autistic disorder and dysmorphic features. Gene. 2012;505(2):384–7. 10.1016/j.gene.2012.05.031 22634100

[45] Gure AO, Wei IJ, Old LJ, Chen YT. The SSX gene family: characterization of 9 complete genes. International journal of cancer Journal international du cancer. 2002;101(5):448–53. 10.1002/ijc.10634 12216073

[46] Lugtenberg D, Zangrande-Vieira L, Kirchhoff M, Whibley AC, Oudakker AR, Kjaergaard S, et al Recurrent deletion of ZNF630 at Xp11.23 is not associated with mental retardation. American journal of medical genetics Part A. 2010;152A(3):638–45. 10.1002/ajmg.a.33292 20186789

[47] Yoshida K, Sugano S. Identification of a novel protocadherin gene (PCDH11) on the human XY homology region in Xq21.3. Genomics. 1999;62(3):540–3. 10.1006/geno.1999.6042 10644456

[48] Veerappa AM, Saldanha M, Padakannaya P, Ramachandra NB. Genome-wide copy number scan identifies disruption of PCDH11X in developmental dyslexia. American journal of medical genetics Part B, Neuropsychiatric genetics: the official publication of the International Society of Psychiatric Genetics. 2013;162B(8):889–97. 10.1002/ajmg.b.32199 24591081

[49] Carrasquillo MM, Zou F, Pankratz VS, Wilcox SL, Ma L, Walker LP, et al Genetic variation in PCDH11X is associated with susceptibility to late-onset Alzheimer's disease. Nature genetics. 2009;41(2):192–8. 10.1038/ng.305 19136949 PMC2873177

[50] Kimberley KW, Morris CA, Hobart HH. BAC-FISH refutes report of an 8p22–8p23.1 inversion or duplication in 8 patients with Kabuki syndrome. BMC medical genetics. 2006;7:46 10.1186/1471-2350-7-46 16709256 PMC1513556

[51] Hollox EJ, Huffmeier U, Zeeuwen PL, Palla R, Lascorz J, Rodijk-Olthuis D, et al Psoriasis is associated with increased beta-defensin genomic copy number. Nature genetics. 2008;40(1):23–5. 10.1038/ng.2007.48 18059266 PMC2447885

[52] Hecquet CM, Zhang M, Mittal M, Vogel SM, Di A, Gao X, et al Cooperative interaction of trp melastatin channel transient receptor potential (TRPM2) with its splice variant TRPM2 short variant is essential for endothelial cell apoptosis. Circulation research. 2014;114(3):469–79. 10.1161/CIRCRESAHA.114.302414 24337049 PMC3978731

[53] Miyake T, Shirakawa H, Kusano A, Sakimoto S, Konno M, Nakagawa T, et al TRPM2 contributes to LPS/IFNgamma-induced production of nitric oxide via the p38/JNK pathway in microglia. Biochemical and biophysical research communications. 2014;444(2):212–7. 10.1016/j.bbrc.2014.01.022 24462864

[54] Miller BA, Hoffman NE, Merali S, Zhang XQ, Wang J, Rajan S, et al TRPM2 channels protect against cardiac ischemia-reperfusion injury: role of mitochondria. The Journal of biological chemistry. 2014;289(11):7615–29. 10.1074/jbc.M113.533851 24492610 PMC3953274

[55] Liu XF, Yu JQ, Dalan R, Liu AQ, Luo KQ. Biological factors in plasma from diabetes mellitus patients enhance hyperglycaemia and pulsatile shear stress-induced endothelial cell apoptosis. Integrative biology: quantitative biosciences from nano to macro. 2014;6(5):511–22. 10.1039/c3ib40265g 24643402

