# Supplementary material for: Novel Loci for Non-Syndromic Coarctation of the Aorta in Sporadic and Familial Cases
Source: PLoS One. 2015 May 18;10(5):e0126873. doi: 10.1371/journal.pone.0126873 (PMC4436177; doi:10.1371/journal.pone.0126873)
Supplement: S1 Table — From all detected CNVs eight with gene content were chosen for validation with MLPA. Only those DNA samples with an aberrant CNV in the particular region were analyzed with a MLPA assay. All CNVs from the microarray analysis could be validated (+), except for the CNV status of the NF1P2 region in eight of 32 samples (duplications instead of deletions); n/a = not applicable because of absent CNV in microarray data. (DOCX) [file pone.0126873.s001.docx]

**S1 Table**

**MLPA validation**

| **Sample** | **GSTM1** | **DEFB** | **NF1P2** | **ERVV1** | **FAM115C** | **SEP T9** | **TPTE** | **TRPM2** |
| --- | --- | --- | --- | --- | --- | --- | --- | --- |
| **10** | + | na | + | + | na | na | na | na |
| **11** | na | na | + | + | na | na | na | na |
| **13** | + | na | na | na | + | na | na | na |
| **14** | na | + | na | + | na | na | na | + |
| **15** | + | na | + | + | na | + | na | + |
| **16** | + | na | + | + | na | na | na | na |
| **17** | + | + | +/– | + | na | na | na | na |
| **18** | na | na | na | + | na | na | na | na |
| **19** | + | na | +/– | + | na | na | na | na |
| **21** | + | + | na | + | na | na | na | na |
| **22** | na | + | na | + | + | na | na | na |
| **23** | na | na | na | na | + | na | na | na |
| **24** | na | na | + | na | na | na | na | na |
| **25** | + | + | na | + | na | na | na | na |
| **26** | + | na | na | + | na | na | na | na |
| **27** | + | na | + | na | na | na | + | na |
| **28** | na | na | na | + | na | na | na | na |
| **29** | + | na | +/– | + | na | na | na | + |
| **30** | + | + | na | + | na | na | na | na |
| **31** | na | + | + | na | na | na | + | na |
| **32** | + | na | na | + | na | na | na | na |
| **33** | na | na | + | na | na | na | na | na |
| **34** | + | na | na | + | na | na | na | na |
| **35** | + | na | + | + | na | na | na | na |
| **37** | na | + | na | + | na | na | na | na |
| **38** | + | na | na | na | na | na | na | na |
| **39** | na | + | na | + | na | na | na | na |
| **40** | + | + | +/– | + | + | na | na | na |
| **41** | + | na | +/– | + | na | na | na | na |
| **42** | na | na | na | + | na | na | + | na |
| **44** | + | + | + | + | + | na | na | na |
| **45** | + | + | na | + | na | na | na | na |
| **46** | + | na | na | + | na | + | + | + |
| **47** | na | na | + | + | + | na | na | na |
| **48** | + | na | na | + | na | na | na | na |
| **49** | + | na | na | + | na | na | na | na |
| **51** | + | na | na | + | na | na | na | na |
| **52** | + | + | +/– | + | na | na | na | na |
| **53** | + | na | + | + | na | na | na | na |
| **55** | + | na | na | na | na | na | na | na |
| **56** | + | + | na | + | na | na | na | na |
| **57** | na | + | na | + | na | + | + | + |
| **58** | na | na | na | + | na | na | na | na |
| **59** | na | na | + | + | na | na | na | na |
| **60** | na | + | + | na | + | na | na | na |
| **61** | na | + | na | + | na | na | na | na |
| **62** | + | + | + | + | + | na | na | na |
| **63** | + | + | na | + | na | na | + | na |
| **64** | na | na | na | + | na | na | na | na |
| **65** | na | na | na | + | na | na | na | na |
| **66** | na | na | + | + | na | na | na | na |
| **67** | na | na | +/– | na | na | na | na | na |
| **68** | + | na | na | + | na | na | na | na |
| **69** | + | na | na | + | na | na | na | na |
| **70** | + | na | na | + | + | na | na | na |
| **71** | na | na | + | + | na | na | na | na |
| **72** | + | na | na | + | na | na | na | na |
| **73** | + | + | + | na | na | na | na | na |
| **74** | na | + | na | + | na | na | na | na |
| **75** | + | + | na | + | na | na | + | na |
| **76** | + | na | na | + | + | na | na | na |
| **77** | na | + | na | + | na | na | na | na |
| **78** | + | na | + | + | na | na | na | na |
| **80** | + | + | na | + | na | na | na | na |
| **81** | na | + | + | na | + | na | na | na |
| **82** | na | + | na | na | na | na | + | na |
| **83** | na | na | na | + | na | + | na | + |
| **84** | + | + | + | + | na | na | na | na |
| **86** | + | + | +/– | + | na | na | na | na |
| **87** | + | na | + | + | na | na | na | na |
| **88** | + | na | na | + | + | na | na | na |
| **# of Tested Samples** | 43 | 36 | 32 | 58 | 19 | 4 | 8 | 6 |

From all detected CNVs eight with gene content were chosen for validation with MLPA. Only those DNA samples with an aberrant CNV in the particular region were analyzed with a MLPA assay. All CNVs from the microarray analysis could be validated (+), exept for the CNV status of the NF1P2 region in eight of 32 samples (duplications instead of deletions); n/a=not aplicable because of absent CNV in microarray data.
